# Applying Farr’s Law to project the drug overdose mortality epidemic in the United States

**DOI:** 10.1186/s40621-014-0031-2

**Published:** 2014-12-10

**Authors:** Salima Darakjy, Joanne E Brady, Charles J DiMaggio, Guohua Li

**Affiliations:** 1 Department of Epidemiology, Columbia University Mailman School of Public Health, 722 West 168th St, New York, 10032 NY USA; 2 Department of Anesthesiology, Columbia University College of Physicians and Surgeons, 622 West 168th St, PH5-505, New York, 10032 NY USA; 3 Center for Injury Epidemiology and Prevention, Columbia University, 722 West 168th St, 5th Floor, New York, 10032 NY USA

**Keywords:** Drug overdose, Epidemics, Farr’s Law, Opioids, Prescription drugs, Epidemiologic methods

## Abstract

**Background:**

Unintentional drug overdose has increased markedly in the past two decades and surpassed motor vehicle crashes as the leading cause of injury mortality in many states. The purpose of this study was to understand the trajectory of the drug overdose epidemic in the United States by applying Farr’s Law. Farr’s “law of epidemics” and the Bregman-Langmuir back calculation method were applied to United States drug overdose mortality data for the years 1980 through 2011 to project the annual death rates from drug overdose from 2012 through 2035.

**Findings:**

From 1980–2011, annual drug overdose mortality increased from 2.7 to 13.2 deaths per 100,000 population. The projected drug overdose mortality would peak in 2016–2017 at 16.1 deaths per 100,000 population and then decline progressively until reaching 1.9 deaths per 100,000 population in 2035.

**Conclusion:**

The projected data based on Farr’s Law suggests that drug overdose mortality in the United States will decline in the coming years and return to the 1980 baseline level approximately by the year 2034.

**Electronic supplementary material:**

The online version of this article (doi:10.1186/s40621-014-0031-2) contains supplementary material, which is available to authorized users.

## Background

In mid-nineteenth century England, William Farr, one of the founders of modern epidemiology, analyzed mortality rates attributed to a smallpox outbreak and derived a “law of epidemics” from the observed patterns, postulating that the dynamics of epidemic outbreaks generally follow a symmetric curve (Farr [1840]). Since that time, the theory that became known as “Farr’s Law” has been applied to projecting the trajectories of the 1885 cattle plague epidemic (Brownlee [1915a]), the 1901 outbreak of smallpox (Brownlee [1915b]), and the AIDS epidemic (Bregman and Langmuir [1990]), with mixed results.

More recently, several authors cited data that demonstrate increases in drug overdose deaths over the past decade (Johnson et al. [2014]; Jones et al. [2013]) or longer (Unick et al. [2013]). Such observations precipitated interventions aimed at limiting opportunities for abuse of controlled substances, particularly prescription opioids. Two of these interventions, the expansion of Prescription Drug Monitoring Programs (PDMPs) across the United States and increased availability and training on rescue treatments that reverse an opioid overdose, have coincided with the stabilization of the annual death rate due to opioid overdoses since 2007 (Brady et al. [2014]). Opioid analgesics account for approximately two thirds of the total drug overdose mortality (CDC [2011]). Temporal trends in drug overdoses closely follow those in prescription drug consumption: as the morphine kilogram equivalents per capita increase, so does the rate of drug overdose mortality. Likewise, both measures began to plateau in 2007 (Brady et al. [2014]). This observation motivated our hypothesis that the drug overdose epidemic is leveling off, and the mortality from drug overdose should start to decline soon and eventually return to a baseline level. Underlying this hypothesis is the fundamental principle of Farr’s Law: epidemics follow a natural sequence in which they spread and subside in a pattern that approximates a symmetric curve (Bregman and Langmuir [1990]). Our objective is to apply Farr’s Law to the drug overdose epidemic and to evaluate over the next several years whether our projections hold true.

## Methods

A drug overdose epidemic curve for the United States was generated by combining observed mortality rates (1980–2011) with mortality rates projected for 2012–2035 following the principles of Farr’s Law. For the observed period (1980–2011), data came from the National Center for Health Statistics (Warner et al. [2011]). Through 1998, unintentional drug overdose deaths were classified using the International Classification of Diseases, Ninth Revision, and included underlying cause of death codes: E850–E858, E950.0–E950.5, E962.0, and E980.0–E980.5. Starting in 1999, deaths were classified using the International Classification of Diseases, Tenth Revision, and included underlying cause of death codes: X40–X44, X60–X64, X85, and Y10–Y14. Crude annual drug poisoning mortality rates were calculated using the annual population for the years observed and the 2011 population for the rates projected for 2012–2035 (CDC WONDER [2014]).

For future projections on the number of drug overdose deaths expected under the principles of Farr’s Law, we applied the method described by Bregman and Langmuir ([1990]) and calculated two ratios: 1) the percent increase in the mortality rate for each year of observed data, and 2) the acceleration—or rate of change—of the first ratio. To fit a symmetric curve, we took the mean acceleration (=0.9876) for the last five years of observed data (2007–2011), and extrapolated the first ratio followed by the number of deaths expected if the principles of Farr’s Law hold for the drug overdose epidemic.

## Findings

**Figure 1:**
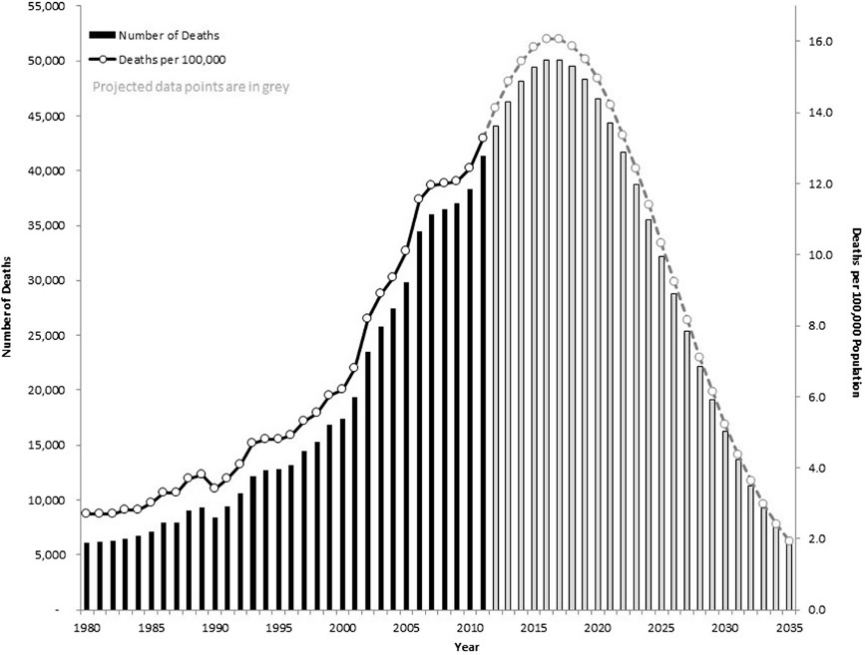
**Annual mortality from drug overdose in the United States 1980–2011, with projections through 2035 based on Farr’s Law (grey data points).**

The annual number of deaths from drug overdoses, along with the percent increase (first ratio) and the acceleration (second ratio) over the previous year are displayed in Table 1. In general, the number of drug overdose deaths in the United States increased progressively from 1980 through 2011. The second ratio indicates that the sharpest annual increases in mortality occurred during the last 10 years of the observed period (in 2000–2001, 2001–2002, and 2005–2006), whereas the relative stability of the first ratio from 2007–2010 indicates a plateau in the number of deaths toward the end of the observed timeframe. Using the mean acceleration for those five years, the projected annual mortality for 2012–2034 steadily declines to 2.4 deaths per 100,000 population, which is similar to the 1980 baseline rate of 2.7 deaths per 100,000 population (Figure 1). Mortality projections for 2011–2035 based on Farr’s Law approximate the right half of a symmetric curve, although our calculations estimate that the rate will decrease from its peak in 2016–2017 to the 1980 baseline level in 18 years, approximately half of the time the rate took to peak.

**Table 1:** **Annual number of deaths and mortality rate per 100,000 population from unintentional drug overdose in the United States, 1980–2011, with projections to 2035**

Year	Deaths ^a^	First Ratio ^b^	Second Ratio ^c^	Deaths per 100,000
1980	6094			2.8
1981	6227	1.0218		2.8
1982	6299	1.0116	0.9900	2.8
1983	6445	1.0232	1.0115	2.8
1984	6723	1.0431	1.0195	2.9
1985	7082	1.0534	1.0098	3.0
1986	7969	1.1252	1.0682	3.3
1987	7920	0.9939	0.8832	3.3
1988	9031	1.1403	1.1473	3.7
1989	9275	1.0270	0.9007	3.7
1990	8413	0.9071	0.8832	3.4
1991	9392	1.1164	1.2308	3.7
1992	10604	1.1290	1.0114	4.1
1993	12133	1.1442	1.0134	4.7
1994	12714	1.0479	0.9158	4.8
1995	12779	1.0051	0.9592	4.8
1996	13227	1.0351	1.0298	4.9
1997	14445	1.0921	1.0551	5.3
1998	15315	1.0602	0.9708	5.5
1999	16849	1.1002	1.0377	6.0
2000	17415	1.0336	0.9395	6.2
2001	19394	1.1136	1.0774	6.8
2002	23518	1.2126	1.0889	8.2
2003	25785	1.0964	0.9041	8.9
2004	27424	1.0636	0.9701	9.4
2005	29813	1.0871	1.0221	10.1
2006	34425	1.1547	1.0622	11.5
2007	36010	1.0460	0.9059	12.0
2008	36450	1.0122	0.9677	12.0
2009	37004	1.0152	1.0029	12.1
2010	38329	1.0358	1.0203	12.4
2011	41340	1.0786	1.0413	13.3
2012	44035	1.0652	0.9876	14.1
2013	46323	1.0520	0.9876	14.9
2014	48127	1.0389	0.9876	15.4
2015	49380	1.0260	0.9876	15.8
2016	50038	1.0133	0.9876	16.1
2017	50076	1.0008	0.9876	16.1
2018	49493	0.9883	0.9876	15.9
2019	48310	0.9761	0.9876	15.5
2020	46570	0.9640	0.9876	14.9
2021	44336	0.9520	0.9876	14.2
2022	41686	0.9402	0.9876	13.4
2023	38709	0.9286	0.9876	12.4
2024	35498	0.9171	0.9876	11.4
2025	32150	0.9057	0.9876	10.3
2026	28757	0.8945	0.9876	9.2
2027	25403	0.8834	0.9876	8.2
2028	22162	0.8724	0.9876	7.1
2029	19095	0.8616	0.9876	6.1
2030	16248	0.8509	0.9876	5.2
2031	13654	0.8404	0.9876	4.4
2032	11332	0.8299	0.9876	3.6
2033	9288	0.8196	0.9876	3.0
2034	7519	0.8095	0.9876	2.4
2035	6011	0.7994	0.9876	1.9

^a^2011 United States population is used to calculate the annual number of deaths projected for 2012–2035.

^b^Percent increase in the mortality rate from the previous year, calculated as deaths in the current year/deaths in the previous year. For example, from 1981 to 1982 the mortality rate increased by a factor of 1.0116 (=6299/6227). Since 1980 is the baseline for this analysis, its first ratio is undefined.

^c^Acceleration (rate of change) of the first ratio, calculated as first ratio for the current year/first ratio for the previous year. For example, from 1981 to 1982 the acceleration in the mortality rate was 0.9900 (=1.0116/1.0218). The second ratios for 1980–1981 are undefined. The second ratio for 2011–2035 is chosen as the mean of the five second ratios for 2007, 2008, 2009, 2010, and 2011.

## Discussion

Our projections based on Farr’s Law indicate that drug overdose mortality in the United States will decrease from its peak in 2016–2017 to the 1980 baseline level in about 18 years. Our projections are made under the assumption that the United States drug overdose epidemic is nearing its peak, as suggested by recent mortality data and opioid prescription data (Brady et al. [2014]; Li et al. [2014]). The shape of the drug overdose mortality curve depends on two key elements: 1) the constant (i.e., the second ratio) chosen for approximating the symmetric curve; and 2) the population count used to calculate the mortality rate. Our decision to use 0.9876 as the second ratio constant was derived from synthesizing information about annual per capita consumption of opioid analgesics and the observed mortality rate accelerations, both of which indicate a shift around 2007 in that opioid sales and deaths stabilized thereafter. The more controversial choice in our projected calculations is the population denominator. Initially, we used the annual population data from the United States Census Bureau in an attempt to factor changes in the number of people at risk. The mortality curve initially generated under the assumption of a dynamic (i.e., increasing) population very closely resembled the one presented in Figure 1. However, since the population susceptible to drug overdose may remain relatively stable in the projected 24-year period, with adults aging both into and out of the highest risk group (45–54 year-olds) (Warner et al. [2011]), we opted for a more conservative method and approximated overdose mortality rates based on the 2011 United States population.

This study represents our first attempt to project the trajectory of the epidemic of drug overdose deaths in the United States. Although this epidemic has been studied extensively through statistical models using predictive variables (Dasgupta et al. [2012]; Li et al. [2014]; Paulozzi and Stier [2010]), we are unaware of any previous study predicting the trajectory of a non-infectious epidemic using Farr’s Law. Our projections, if partially accurate, may help assess intermediate outcomes to gauge whether interventions are working and guide long-term planning and management of public health resources and prevention efforts. When access to prescription medication becomes more difficult with enforcement of PDMPs, there is a hazard of diversion whereby people shift to using alternative substances such as heroin. To avoid this substitution effect would require multifaceted interventions such as the expansion of prescription limits, increased drug screening, requiring pain contracts, and the distribution of naloxone to first responders.

There are several limitations to our study. First, this is a crude approximation of an epidemic curve based on assumptions that guided our choices of the acceleration constant and population at risk. Although the method we applied originated from studies of infectious diseases, it is unknown whether Farr’s Law applies to epidemics of a non-infectious origin. It is plausible that a non-communicable disease, such as drug overdose, can follow infectious patterns. Indeed, a theory of social contagion has been proposed for a myriad of behavioral disorders (Eisenberg et al. [2013]) as well as obesity (Christakis and Fowler [2007]), albeit with some controversy in the latter example. As with behavioral disorders, the social mechanism of transmission may underlie drug-use initiation, continuation, and transition such that those activities then mimic infectious disease patterns, proliferating through the population until some natural threshold or intervention prevents further spread. Second, for simplicity, we estimated drug overdose mortality for the projection period without considering changes in the size of the population or the age distribution of the population. This may bias our estimates somewhat because the United States population is projected to gradually grow in size and get older in age composition. Furthermore, these results are limited to overdose mortality data and are for the United States as a whole. Thus, the results may not reflect trends in non-fatal drug overdose incidents and in specific geographic areas. Despite these limitations, the predicted epidemic curve guided by Farr’s Law suggests that annual drug overdose mortality is likely to decline in the coming years and probably return to the baseline level by the year 2034.

## Conclusions

Mortality data over the next two decades will ultimately test the accuracy of our projections. If the drug overdose epidemic is indeed waning, it may imply that the intensified efforts in recent years, such as enhanced prescription drug monitoring, are working and should be continued.

## Author contributions

SD reviewed the literature, analyzed the data, and wrote the first draft of the manuscript. JEB acquired the data, supervised the data analysis, and contributed to the critical revision of the manuscript. CJD contributed to the study design, statistical approach, and critical revision of the manuscript. GL conceived of the study, secured funding, oversaw the implementation of the research plan, and contributed to the critical revision of the manuscript. All authors read and approved the final manuscript.

## Authors’ original submitted files for images

Below are the links to the authors’ original submitted files for images. Authors’ original file for figure 1
