# Supplementary figures and images for: Applying Farr’s Law to project the drug overdose mortality epidemic in the United States
Source: Inj Epidemiol. 2014 Dec 10;1(1):31. doi: 10.1186/s40621-014-0031-2 (PMC5005643; doi:10.1186/s40621-014-0031-2)

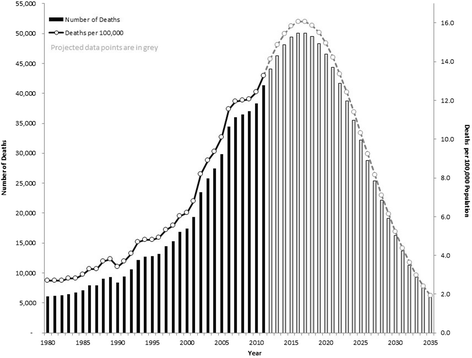

Supplement: Supplementary file 1 — Authors’ original file for figure 1 [file 40621_2014_31_MOESM1_ESM.gif]
